# An era of single-cell genomics consortia

**DOI:** 10.1038/s12276-020-0409-x

**Published:** 2020-09-15

**Authors:** Yoshinari Ando, Andrew Tae-Jun Kwon, Jay W. Shin

**Affiliations:** RIKEN Center for Integrative Medical Sciences, 1-7-22 Suehiro-Cho, Tsurumi-Ku, Yokohama, 230-0045 Japan

**Keywords:** Data integration, Genetics research

## Abstract

The human body consists of 37 trillion single cells represented by over 50 organs that are stitched together to make us who we are, yet we still have very little understanding about the basic units of our body: what cell types and states make up our organs both compositionally and spatially. Previous efforts to profile a wide range of human cell types have been attempted by the FANTOM and GTEx consortia. Now, with the advancement in genomic technologies, profiling the human body at single-cell resolution is possible and will generate an unprecedented wealth of data that will accelerate basic and clinical research with tangible applications to future medicine. To date, several major organs have been profiled, but the challenges lie in ways to integrate single-cell genomics data in a meaningful way. In recent years, several consortia have begun to introduce harmonization and equity in data collection and analysis. Herein, we introduce existing and nascent single-cell genomics consortia, and present benefits to necessitate single-cell genomic consortia in a regional environment to achieve the universal human cell reference dataset.

## Genomics: Collaborative efforts to map the human body—single cell at a time

Profiling the human body at the single cell resolution will reveal a map to enable researchers to compare cell types that are found in healthy and disease tissues at a much finer resolution. Large international projects such as FANTOM (Functional ANnoTation Of the Mammalian genome) and GTEx (Genotype-Tissue Expression) have profiled gene expression in hundreds of human cell types. However, the data were obtained from ‘bulk’ samples. The possibility of sequencing large numbers of single cells at a reasonable cost offers the opportunity to create a much more detailed human cell reference map. Jay Shin, at the RIKEN Institute, Yokohama, Japan, and colleagues review rapidly growing single cell genomics consortia and highlight the advantages of initiating national collaborations to harmonize procedures and to obtain a more accurate representation of regional ethnic diversity.

## Introduction

RIKEN has spearheaded international consortium efforts to establish an atlas of human promoters and enhancers through the FANTOM project. Gene expression profiling of 400+ human cell types using CAGE revealed gene-regulatory modules that define cell types and states^1,2^. This comprehensive landscape resource has revealed pervasive transcription of coding and noncoding RNA in the human genome, and precise understanding of how and where genes are activated^3^. However, the FANTOM data were derived from bulk samples, ignoring the cellular heterogeneity that exists in both tissues and the cell culture system. Creating an atlas that maps promoters and enhancers across millions of single cells in the human body will not only reveal regulatory regions of our genome, but also gene-regulatory programs that control cell fates and pathology of genetic diseases.

Similarly, the genotype-tissue expression (GTEx) project has generated a large genomic dataset, including over 10,000 bulk RNA-seq samples representing 54 different tissues (30 organs) acquired from 948 individuals with genotype information^4–10^. This rich dataset allows for linking genetic variants at gene expression levels through expression quantitative trait loci analysis (eQTL). Despite its efforts to collect a variety of tissues from a relatively large cohort of individuals, the expression profiles are based on bulk, lacking cellular heterogeneity. To circumvent this, the GTEx project has recently released a unique strategy to infer cellular heterogeneity based on gene signatures from different cell types known to be present in a given tissue. The method relies on the *Tabula Muris* dataset^11^ to deconvolute the cellular composition over 6,000 additional GTEx samples corresponding to 28 tissues, and reveals tissue-specific eQTLs colocalizing with GWAS variants that were not detected in bulk, but only discovered through deconvolution strategy^12^. GTEx has built an extensive and mature infrastructure to obtain fresh tissues from relatively large cohorts. It is a matter of time before the consortium combines new technologies such as single-cell RNA-seq with archival and new tissues for single-cell eQTL RNA-seq analysis^13^.

Thanks to recent technological advances, we can now profile large numbers of dissociated cells, and study the RNA transcripts, proteins, and chromatin profiles of 10–100 k individual cells at a reasonable cost (*consensus approach*). Moreover, we can characterize DNA sequences for reconstruction of cell lineages^14^, and combine these to relate different gene features to cellular identities. We can also profile multiple classes of RNA, including noncoding RNAs, enhancer RNAs^15^, and multiplex transcripts and proteins in situ to map cells and their molecules to their positions in histological sections (*spatial approach*)^16–19^. Applying both consensus and spatial strategies across tissues and populations, together with advanced database infrastructure and computational tools, should allow us to define “what is normal” in cells, and provide a universal reference map of the human body.

In recent years, numerous reports demonstrating the power of single-cell genomics are prevailing, where topics include cellular ontology^20,21^ and functional conservation across species^22^; cell fate and lineage determinants^23,24^; dynamic changes in cell states such as the cell cycle^25^ and transient responses^26^; molecular mechanisms that control intra- and intercellular regulatory networks^27,28^; fundamental research in disease studies and pathology^29–32^. In parallel, tens and hundreds of thousands of single-cell genomics data across various human tissues are leading to the discovery of new cell types and states, fundamentally changing the picture of human anatomy in multiple ways (summary of large-scale single-cell genomics data across human tissue in Table 1). Integrating our knowledge that we gained through single-cell genomics shows tremendous potential for translational discoveries and applications, and impacting diagnostic and clinical practices.

**Table 1:** Single-cell profiling of major human organs and tissues.

Systems	Organs/tissues	Notes	References
Hematopoietic and immune system	Blood	Discovery of new types of blood cells	^95^
		Hematopoietic differentiation	^96^
		Immune cell atlas (review)	^97^
Nervous system	Brain	Transcriptional and epigenetic states	^98^
		Human and mouse cortex	^51^
		Alzheimer’s disease	^31,32^
		Autism	^99^
Urinary system	Kidney	Single-nucleus RNA-seq pipeline	^100^
		Lupus nephritis	^101^
		Spatiotemporal immune topology	^102^
Respiratory system	Lung	Lung cell atlas (review)	^103^
		Asthma	^104^
		Pulmonary fibrosis	^105^
	Trachea	Discovery of pulmonary “ionocytes”	^106^
Hepatopancreatic–biliary	Liver	Liver cell atlas	^107^
	Pancreas	Pancreas cell atlas	^108^
Gastrointestinal system	Colon	Ulcerative colitis	^109^
	Small intestine	Crohn’s disease	^110^
Cardiovascular system	Heart	Stress-related lincRNA	^111^
Developmental biology	Fetal kidney	Progenitor cell dynamics and lineage	^112^
	Fetal liver	Fetal liver hematopoiesis	^113^
	Fetal heart	Autoimmune-associated congenital heart block	^114^
	Maternal–fetal interface	Trophoblast–decidual interactions	^27^
Pediatric		Pediatric cell atlas (review)	^115^
Reproductive system	Breast	Breast epithelial cells	^116^
	Testis	Testis cell atlas	^117^
Sensory system	Eye	Retina cell atlas	^118,119^

Cells in our body can now be explained by several features, including their shape, location in a tissue, gene expression, and function in a high-throughput manner. However, we have not comprehensively determined how these features are associated with each other, and what constitutes “normal” with respect to the health status of an individual. As a result, our knowledge of the cellular makeup and relations of the human body and disease is still limited. Therefore, we need a comprehensive reference database through an integrative, systematic effort, and many teams of scientists working together to produce data that are not only consistent, high quality, and interoperable, but also driven by biology and medicine.

In the last few years, several single-cell genomics consortia have been created to address the issue of systematic data integration and harmonization. Consortia such as the Human Cell Atlas aims to bring together domain experts to consolidate single-cell data in a central portal. In parallel, several tissue-centric consortia, such as the BRAIN initiatives, aim to dive deeper into the complex nature of individual organ systems. Here, we summarize the ever-growing single-cell genomics consortia and describe their missions. We further showcase benefits from generating single-cell data in a regional and coherent manner through the formation of single-cell consortia.

## Single-cell genomics consortia

In light of the enormous complexity of the human body and the rapidly evolving technology landscape, in October 2016, more than 150 international scientists met in London to launch the planning process for an ambitious new initiative: the Human Cell Atlas (HCA), an international collaboration to create comprehensive reference maps of all human cells^33–36^. The HCA consortium aims to build this ambitious yet essential resource in phases, starting with cells in tissues and eventually organs and systems, with the aim of constructing an increasingly detailed, valuable, and comprehensive atlas with guiding principles for the community that includes open data sharing high-quality data, equity, ethical considerations, flexibility, international, and technology and computational innovations^37^.

At the time of writing, hundreds of thousand single-cell data across major organ tissues, including colon, liver, immune, and developmental tissues, already populated the database^38^. The recent meeting in Barcelona, Spain, laid out the roadmap toward building the first draft of the atlas, where the draft will clearly define not only cell types but also cell states, identify the molecular program in both dissociated and spatial contexts, and project the trajectory and relation to time. The first draft of the HCA aims to profile the molecular and spatial characteristics of cells from major tissues and systems from healthy donors, with geographic, age, and ethnic diversity in mind.

The unique nature of the HCA is forming a large global coalition of scientists that builds upon grassroot communities to work toward a common goal. The support of seed funds from philanthropic foundations and government bodies jumpstarted the project, connecting scientists all around the globe. However, with membership reaching over 1600 and over 100 research institutes represented, arriving at a single solution to create the atlas can be logistically challenging to coordinate. At the same time, the relatively flexible, community-based nature of HCA attracts motivated scientists to freely explore the field and collaboratively construct platforms that will set new standards for building an international reference for human cells.

Concurrently, several government and philanthropic-led programs are in full swing that are incentivized through defined funding structures. In 2003, a Swedish-based program called the Human Protein Atlas aimed to map all human proteins in cells, tissues, and organs using integration of various omics technologies, including antibody-based imaging and RNA-seq. Their current Tissue Atlas includes 44 normal human tissue types with 15,313 genes represented in the protein data with available antibodies^39^. Concurrent programs include The Cell Atlas, which represents 64 cell lines with subcellular details^40^, The Pathology Atlas representing 17 different cancer types^41^, The Brain Atlas of the human, pig, and mouse, The Blood Atlas including the secretome, and The Metabolic Atlas with over 120 curated molecular pathways. Their latest efforts to consolidate data with FANTOM5 and GTEx RNA gene expression data aim to reach a multi-omics integrated portal system^42,43^. In the European Union (EU), the LifeTime initiative was recently introduced to fundamentally impact basic science across multiple fields, including developmental biology, regeneration, and stem cell biology through single-cell genomic technologies^44^. With strong emphasis on disease and collaboration with industry partners, the initiative aims to synthesize novel solutions based on single-cell genomics technologies to improve human health and reduce the economic burdens of the aging population.

In the United States, the National Institutes of Health (NIH) have recently reported to support the Human Biomolecular Atlas Program (HuBMAP)^45^ for 7 years. The consortium aims to develop a widely accessible framework for mapping the human body at single-cell resolution, with a strong focus on spatial molecular mapping. Unlike GTEx, HuBMAP focuses on generating single-cell data using samples from a more limited number of individuals while investing in a robust common coordinated framework to make data more integratable and communicable across various consortia.

## Tissue-centric consortia

A broad profiling of the human body across many tissues will certainly be necessary to understand the holistic anatomy of our body. At the same time, the intricacies of individual organ systems and their associated diseases are unending. Several disease- or tissue-centric consortia are starting to adapt single-cell genomics to dive deeper into resolving the map of each organ system. In December 2016, the US Congress authorized $1.8 billion in funding for the Cancer Moonshot over 7 years, covering a wide range of areas, including patient engagement, drug resistance, prevention, and early detection of hereditary cancer^46^. One of the flagship projects includes the human tumor cell atlas, a collaborative project to build three-dimensional atlases of cellular, morphological, and molecular features of human cancer over time. The consortium is organized with a central data coordination center working with the human tumor atlas focusing on advanced cancers, and the pre-cancer atlas (PCA) focusing on conditions that are likely to become cancer. Comparative datasets from healthy counterparts, such as the human cell atlas, may become essential to interpret the risks and severity of diseases such as cancer.

As one of the BRAIN initiative’s priority areas, the consortium aims to characterize all cell types in the nervous system at single-cell resolution^47^, and to develop tools to record, mark, and manipulate neurons in the living brain. Comparing human and nonhuman primates, the group previously revealed global, regional, and cell-type-specific species expression differences in rare subpallial-derived interneurons expressing dopamine biosynthesis genes in humans^48^. More recently, the group performed single-cell RNA-seq on 40,000 cells to create a high-resolution single-cell gene expression atlas of the developing human cortex^49^, permitting inference of gene-regulatory networks involved in neurogenesis, evolution, and neuropsychiatric diseases. Similarly, the Allen Brain Atlas, led by the Allen Brain Institute, for many years, has driven large-scale mapping projects in the brain^50^. Seeking to combine genomics with neuroanatomy by creating gene expression maps for the mouse and human brain, they recently used single-cell SMART-seq analysis to profile 50,000+ cells across the human and mouse cortex. In their recent work, they identified a highly diverse set of excitatory and inhibitory neurons that are mostly sparse, and showed high conservation in cellular programs. At the same time, the authors reported stark differences in cellular proportions, laminar distributions, gene expression, and morphology between humans and mice^51^.

LungMAP: The Molecular Atlas of Lung Development Program^52,53^ is a NIH-funded consortium focusing on the human lung that serves as a research resource and public education tool. The consortium of four research centers, a data-coordinating center, and a human tissue repository integrates imaging, transcriptomics, and proteomics in a comprehensive data resource called BREATH. The group recently published comprehensive anatomic ontologies for lung development, comparing alveolar formation and maturation within mouse and human lung^54^. Cellular ontology is an important step toward standardizing and expanding the current terminology of fetal and adult lungs as a resource for broader single-cell genomics consortia.

Other tissue-centric consortia include the Kidney Precision Medicine Program (KPMP) that aims to create a kidney tissue atlas^55^, the Immunological Genome Project (ImmGen), where they recently published a matched epigenome and transcriptome analysis in 86 primary cell types spanning the mouse immune system, establishing an atlas of 512,595 active *cis*-regulatory elements^56,57^, and the GenitoUrinary Development Molecular Anatomy Project (GUDMAP), where they focus on spatial imaging and gene expression profiling of the kidney, lower urinary tract and nociceptors (pain receptors), and the associated cell types in pain processing of the urinary and pelvic regions in mice and more recently in human samples^58,59^. The list of single-cell genomics consortia is growing and is summarized in Table 2.

**Table 2:** List of existing and nascent single-cell genomics consortiums.

Consortium	Short name	Mission	Website	Funding source
The Human Cell Atlas	HCA	To create comprehensive reference maps of all human cells as a basis for both understanding human health and diagnosing, monitoring, and treating disease	https://www.humancellatlas.org/	Various^a^
Human Biomolecular Atlas Program	HuMAP	To develop an open and global platform to map healthy cells in the human body	https://hubmapconsortium.org/	NIH
Gene-Tissue Expression	GTEx	To build a comprehensive public resource to study tissue-specific gene expression and regulation	https://gtexportal.org/	NIH
LifeTime Initiative	LifeTime	Revolutionizing healthcare by tracking, understanding, and treating human cells during diseases	https://lifetime-fetflagship.eu/	EU (Horizon 2020)
The Human Protein Atlas	HPA	To map all the human proteins in cells, tissues, and organs using integration of various omics technologies, including antibody-based imaging, mass spectrometry-based proteomics, transcriptomics, and systems biology	https://www.proteinatlas.org/	Knut and Alice Wallenberg Foundation (main)
Single-cell eQTLGen Consortium	sc-eQTLGen	To identify the upstream interactors and downstream consequences of trait-related genetic variants in specific immune cell types	https://www.eqtlgen.org/single-cell.html	Various
The Human Tumor Cell Atlas	HTCA	Constructing three-dimensional atlases of the cellular, morphological, and molecular features of human cancers over time	https://humantumoratlas.org/	NIH/NCI
Allen Brain Atlas	Allen Brain Map	Accelerating progress toward understanding the brain	https://portal.brain-map.org/	Philanthropic (Paul G. Allen)
The BRAIN initiative	Cell Census Network (BICCN)	Discovering diversity: identify and provide experimental access to the different brain cell types to determine their roles in health and disease	https://braininitiative.nih.gov/	NIH
The Molecular Atlas of Lung Development Program	LungMAP	Omics, images, 3D viewer, and Analytics platform to study the human lung	https://lungmap.net/	NHLBI
Kidney Precision Medicine Program	KPMP	Purpose of understanding and finding new ways to treat chronic kidney disease (CKD) and acute kidney injury (AKI)	https://kpmp.org/	NIDDK
Immunological Genome Project	ImmGen	To computationally reconstruct the gene- regulatory network in immune cells	http://www.immgen.org/	NIAID
GenitoUrinary Development Molecular Anatomy Project	GUDMAP	Focused on the kidney, lower urinary tract, and nociceptors (pain receptors), and expanding our understanding of human development of the genito-urinary system	https://www.gudmap.org/	NIDDK

*NIH* National Institute of Health, *NHLBI* National Heart, Lung, and Blood Institute, *NIDDK* National Institute of Diabetes and Digestive Kidney Diseases, *NIAID* National Institute of Allergy and Infectious Diseases.

Various: Supported by individual funds.

^a^Infrastructure support by Chan Zuckerberg Initiative (CZI) https://chanzuckerberg.com/.

## Building a single-cell genomics consortium

Despite international efforts to integrate single-cell genomics data, such as the HCA, establishing a consortium in a local environment will benefit science in multiple ways: (1) research and clinical networks within the respective nations spark a new level of scientific collaboration that builds toward clinical and translational research; (2) physical proximity suggests easier access to samples that often leads to manageable coordination toward standardization, tissue procurement, and minimizing batch effects; (3) data from local cohorts generate appropriate genetic and environmental backgrounds with key emphases on diseases that are prevalent in the region; (4) empowers local scientists toward genomics technology and computational innovations; and (5) directly addresses local regulations and policies around ethics and data sharing. Here, we exemplify, non-exhaustively, our efforts in Japan toward building a regional single-cell genomics consortium.

### Systematic workflows

**Fig. 1:**
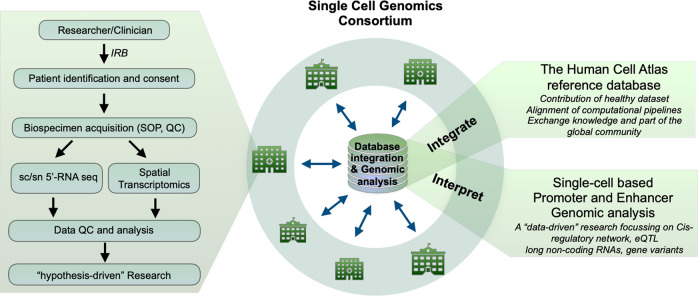
A model of single cell medical network (SC_MED) consortium. A systematic workflow to facilitate each collaborating partner through IRB, sample acquisition, technology profilling and data analysis to realize publication. In parallel, seamless integration of data from all collabarators to contribute healthy samples to the human cells atlas database. Genomics analysis based on 5-based technology to inter Cis-gene regulatory network across the human body.

The single-cell genomics consortium by nature unites scientists and clinicians from different disciplines to spur cross-disciplinary creativity while providing the necessary structure to guide the effort. To better standardize and coordinate efforts from sample collection and data production to analysis, we need to establish a systematic workflow to coordinate with clinicians and researchers across Japan, involving sample-processing standard operating protocols (SOPs), quality control (QC) metrics, central databases, analytical pipelines, and ethics and data policies (Fig. 1).

The single-cell genomics consortium in Japan aims to generate single-cell datasets, mostly with standardized 5′-RNA-seq technology, derived from both healthy and disease samples that will incubate within the consortium, while data generated from healthy samples will be shared with the global HCA community as early as possible. We will also incorporate HCA data, both cellular and spatial, as well as key analytical pipelines, and apply them to address biology-driven questions posed by individual biological collaborators. In parallel, we will integrate 5′-based single-cell data from multiple sample providers to explore gene regulation, focusing on promoter and enhancer activities, on a global scale, leading to the *cis*-regulatory atlas.

To ensure high-quality single-cell data production, the consortium created a central data center in RIKEN and a team of sample coordinators that closely interacts with individual sample providers to optimize protocols and QC for library production and sequencing. Most human specimens will come from clinical biopsies and surgical resections of living patients, and occasionally from healthy living donors, deceased transplant organ donors, and rapid autopsy from deceased donors. Therefore, maximizing sample quality early in sample collection by minimizing the time between biopsy/resection and preservation is critical. Although several reviews comparing dissociation methods have been reported^60–62^, the “gold standard” SOP for tissue dissociation, unfortunately, is not available at this stage. When access to fresh samples is not possible, cryopreservation after cellular dissociation may maintain higher quality compared with direct cryopreservation of the whole biospecimen. Nonetheless, cryopreservation of adjacent sections or tissues will benefit by gathering pathological information and storage for future technologies, and implementing complementary methods, such as multiplexed spatial analysis, should reflect cellular compositions found through dissociation methods. To further minimize possible technical and biological variations, the consortium can provide SOPs with clear instructions, and requires comprehensive metadata (donor information, site, and time). The Human Cell Atlas relies on a central repository for SOPs (protocols.io^63^) and systematic collection of metadata. Constant exchange of protocols and metadata with the open-source community will move toward standardization in the long run.

Performing cell sorting to enrich the desired cell type is possible, although conventional fluorescence-activated cell sorters (FACS) contain insidious chemicals and induce physical stress to cells that may alter gene expression profiles. The latest single-cell genomics platforms can profile a relatively large number of cells (~3000–5000 cells) in a single run, allowing for unbiased sampling of the cell population without FACS. When the desired cell population is rare, performing negative selection by means of bead-conjugated antibodies targeting unwanted cell populations (e.g., dead cells and CD45+ immune cells) will significantly enrich for target cells, and at the same time, minimize cellular stress. Concurrently, DNA-barcoded antibodies can be used to target specific epitopes and profile the transcriptome and target protein^64^.

The consortium requires robust QC metrics that are critical to the success of downstream processes. A high proportion and appropriate number of viable cells will increase the chance of generating a high-quality dataset. Additional metrics to avoid sample mislabeling, patient data swapping, and employing robust computational QCs are implemented to ease data integration and lead to a more biologically meaningful interpretation of single-cell genomics data.

### Data integration and genomic analysis

Compared with bulk data analysis, single-cell genomics data bring unique challenges in their analysis in two aspects: high dimensionality due to the sheer increase in the number of observations made, and high variability from the inherent sparsity of the data stemming from both biological variations and limited sensitivity of the current methods. Furthermore, the massive amount of data that are generated from single-cell analyses brings additional challenges in data access, management, and infrastructure. As such, we established a robust database framework to handle vigorous activities that are specifically tailored to address individual collaborators while maintaining standardization through single-cell genomics platforms, dissociation protocols, centralized databases, and experimental designs. The general outline of computational tools is described below; however, to narrow the gap between computational scientists and sample collaborators, the consortium continues to develop and implement graphic user interfaces that can be easily implemented by collaborating members.

Estimation of the gene expression levels from scRNAseq data requires careful quality control steps to remove unwanted noise from cell debris or free-floating RNA. The raw expression data that pass this QC step need to be normalized using single-cell-specific approaches, such as the use of spike-ins and modeling of cell-specific factors, as the global scaling approach used in bulk data analysis is no longer suitable [CellRanger^65^ and SCATER^66^]. A number of expression-level imputation approaches have been developed as well to estimate the expression values that might have been missed owing to dropout events [MAGIC^67^, scIMPUTE^68^, and SCRABBLE^69^]. In addition, the data need to be corrected for other confounding factors such as batch effects and cell cycles [SEURAT^70^, fastMNN^71^, SCLVM^72^, and CCREMOVER^73^].

Once the data processing is complete, the next step is to assign identities to individual cells, which are usually from mixed populations. This step generally involves dimensional reduction and clustering of the expression data to group the cells with similar transcription profiles [PCA^74^, TSNE^75^, and UMAP^76^]. While traditional clustering methods, such as hierarchical clustering, can be used, a number of single-cell-specific methods have been developed, and there are benchmarks [SC3^77^ and DUO^78^]. For those cells undergoing continuous differentiation or stimulations, trajectory inference techniques have been used to assign them onto a continuous path of changes in order to establish a temporal ordering of the cells, which is referred to as pseudotime [DIFFMAP^79^, MONOCLE^80^, and RNAVELOCITY^81^]. Finally, when discussing cell identity, one confounding factor that is especially relevant to single-cell genomics consortia is how one differentiates between cell type (stable features of a cell’s identity) and cell state (transient aspects of a cell’s status). How to firmly establish these concepts using data-driven and generalizable approaches is a discussion that is necessary within the single-cell consortium.

Once the cell types have been established, we can proceed to identify the gene signatures that are specific to each type, and make inferences about the biology behind them. The most common technique is to perform differential expression analysis among different populations of cell types or states. Due to the technical challenges imposed by the high dispersion and dropout events inherent in scRNAseq data, numerous efforts have been made to develop single-cell-specific techniques that address these issues [MAST^82^ and SCDE^83^]. Other approaches involve the inference of gene-regulatory networks, using both existing methods developed for bulk data and newly developed single-cell-specific methods [WGCNA^84^ and SCODE^85^].

Finally, the ongoing efforts to generate single-cell genomics data that can be spatially resolved have brought some important advances recently, giving us a chance to not only identify the cell types but also their spatial locations in the original tissue the cells were sampled from [STAHL^86^, SEQFISH^18^, and SLIDESEQ^19^]. This has important implications for single-cell genomic consortia, as it will allow us to investigate the interplay between gene-regulatory networks and physical locations of different cell types that interact with one another. For readers who would like more in-depth reviews of the current methodologies employed in this field, there are a number of comprehensive review papers and handbooks that have been published in recent years^87–90^.

## Perspective

Despite the generation of a growing amount of single-cell data, single-cell consortia from the US/EU can inadvertently lead to biased representation of single-cell genomics data, further exacerbating the skewness of genetic representation that was pandemic during the genomic era^91,92^. For instance, a concern for lack of Asian genomes in the reference datasets is rising (e.g., 1.3% of GTEX is Asian, where 59.6% of Asians make up the world’s population^93^). Poor representation in reference data can lead to misinterpretation of research and clinical results^94^. While greater efforts are being made to represent regional/ethnic diversity in global consortia including HCA, uplifting regional research groups to lead data production is necessary, and creation of a local consortium can be one of the steps to achieve meaningful human cell reference for all. It will be imperative to work with regional research–clinical communities together with funding agencies to initiate a dialogue toward better standardization and harmonization of single-cell genomics data while maintaining a constructive relationship with global single-cell genomics communities to engage and represent all of us on the global scale.
